# Allergen-specific immunotherapy provides immediate, long-term and preventive clinical effects in children and adults: the effects of immunotherapy can be categorised by level of benefit -the centenary of allergen specific subcutaneous immunotherapy

**DOI:** 10.1186/2045-7022-2-8

**Published:** 2012-04-13

**Authors:** Lars Jacobsen, Ulrich Wahn, M Beatrice Bilo

**Affiliations:** 1 Research Centre for Prevention and Health, Glostrup University Hospital, Copenhagen, Denmark; 2 Allergy Learning and Consulting, Krokushaven 18, DK 2765 Smorum, Copenhagen, Denmark; 3 Children's Hospital Charité, Virchow Klinikum, Berlin, Germany; 4 Allergy Unit, Department Immunology, Allergy and Respiratory Diseases, University Hospital, Ancona, Italy

**Keywords:** Allergy, Rhinitis, Asthma, Specific immunotherapy, Immunological mechanisms, Antibodies, IgG, IgE, Long-term effect, Prevention

## Abstract

Allergen Specific Immunotherapy (SIT) for respiratory allergic diseases is able to significantly improve symptoms as well as reduce the need for symptomatic medication, but SIT also has the capacity for long-term clinical effects and plays a protective role against the development of further allergies and symptoms. The treatment acts on basic immunological mechanisms, and has the potential to change the pathological allergic immune response. In this paper we discuss some of the most important achievements in the documentation of the benefits of immunotherapy, over the last 2 decades, which have marked a period of extensive research on the clinical effects and immunological background of the mechanisms involved. The outcome of immunotherapy is described as different levels of benefit from early reduction in symptoms over progressive clinical effects during treatment to long-term effects after discontinuation of the treatment and prevention of asthma. The efficacy of SIT increases the longer it is continued and immunological changes lead to potential long-term benefits. SIT alone and not the symptomatic treatment nor other avoidance measures has so far been documented as the therapy with long-term or preventive potential. The allergic condition is driven by a subset of T-helper lymphocytes (Th2), which are characterised by the production of cytokines like IL-4, and IL-5. Immunological changes following SIT lead to potential curative effects. One mechanism whereby immunotherapy suppresses the allergic response is through increased production of IgG4 antibodies. Induction of specific IgG4 is able to influence the allergic response in different ways and is related to immunological effector mechanisms, also responsible for the reduced late phase hyperreactivity and ongoing allergic inflammation. SIT is the only treatment which interferes with the basic pathophysiological mechanisms of the allergic disease, thereby creating the potential for changes in the long-term prognosis of respiratory allergy. SIT should not only be recognised as first-line therapeutic treatment for allergic rhinoconjunctivitis but also as secondary preventive treatment for respiratory allergic diseases.

## Introduction

Allergy is a systemic disease with a local response following allergen exposure. Rhinitis, asthma and Bronchial Hyperresponsiveness (BHR) are closely related and a systemic pathway, involving the bloodstream and bone marrow, contributing to the cross-talk between the upper and lower airways [1].

The close relationship between allergic rhinitis and allergic asthma and the co-morbidity of upper and lower airway diseases has been carefully described elsewhere [2-4]. In asthmatic children it has been shown that asthma was more severe when in concomitance with allergic rhinitis and that increased asthma medication was required [5]. In asthmatic adults with concomitant allergic rhinitis asthma control is less manageable while there is a higher rate of asthma exacerbations and emergency room visits [6]. Allergic rhinitis is a major risk factor for the later development of asthma [3,7] and more than 20% of all rhinitis patients develop asthma later on in life [8]. Up to 50% of rhinitis patients have increased bronchial hyperresponsiveness during, as well as outside the pollen season [9] and an ongoing subclinical level of inflammation [10]. Allergic sensitivities usually increase with age [11] and being sensitised to one allergen source also increases the risk for developing new sensitizations over time [12]. The understanding of allergy as a chronic systemic immunological condition should be the platform for the choice of diagnostic, treatment as well as monitoring options in the allergic patient.

Allergen specific immunotherapy (SIT) produces a decrease in symptoms and in the need for medication, but SIT also has the capacity for long-term clinical effects and for the prevention of the development of further allergies and symptoms. The treatment acts on the basic immunological mechanisms responsible for causing symptoms and has the potential to change the immune response and the pathological pathways responsible for the allergic symptoms [13]. SIT is an anti-inflammatory, causal and preventive treatment for respiratory allergic diseases [14].

The year 2011 marked the 100th anniversary of the first publication on SIT by Leonard Noon [15]. Since then, SIT has been widely used in the treatment of respiratory allergic diseases - at first on a more or less empirical basis. For a number of years the treatment has progressively evolved along with greater medical and immunological insights. Basic immunological research, specific knowledge about allergic diseases and intensive research related to the allergenic molecules and the extracts used for diagnosis and therapy have been continuously developed and as a result improved the opportunities for specific treatment of respiratory allergic diseases [16,17]. More recently the regulatory environment has evolved and allergen extracts are regarded as pharmaceutical specialties with an increased need and request for documentation and clinical data on effectiveness and safety [18,19].

This paper highlights and examines some of the most important developments and information regarding the potential benefits of SIT achieved over the last 10-20 years - a period marked by much clinical activity, explorative as well as confirmative studies on SIT and the publication of international guidelines to support clinical practice [4,20-22]. Although the use of sublingual immunotherapy (SLIT) with the commercial availability of new products represents the most recent and well documented form for immunotherapy, the majority of explorative studies on basic immunology, mechanisms of application of allergen products and long-term clinical potential have been performed using the original subcutaneous immunotherapy (SCIT) treatment concept. All these studies which have been performed mainly based on scientific interest, have established a very important basis for the design of guidelines for production of the basic clinical documentation on efficacy and safety of new product. It is of paramount importance that in the new wider documentary immunotherapy studies we also seek confirmation of the knowledge we have achieved over the many years of investigator driven data exploration.

### SIT - mechanism of action

Different immunological effector cells are responsible for allergic inflammation [23-25]. The allergic disease is driven by a subset of T-helper lymphocytes (Th2), which are characterised by the production of cytokines like IL-4, IL-5 and IL-13 among others. These cells and their cytokines are responsible for the effects on other cells involved in the allergic response. The most important effector cells in the allergic immunological response are eosinophils, mast cells and basophils. Th2 cells play an essential role in the promotion of allergen-specific IgE synthesis by B cells.

**Figure 1:**
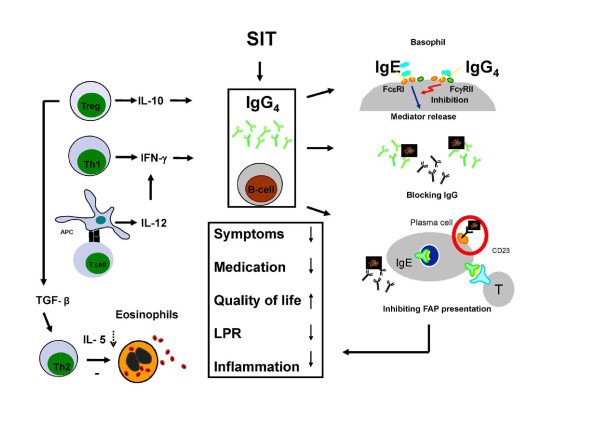
**The immunological response to allergen specific immunotherapy**. The immunological response to allergen specific immunotherapy.

The benefits and the clinical effects of SIT should be evaluated and analysed in terms of the immunological changes that follow the treatment. SIT acts by influencing basic immunological mechanisms [26] resulting in the suppression of the seasonal increase in eosinophilia [27], in reduction of the late-phase reactivity [28,29] and a shift from a Th2- to Th1-like response is initiated and maintained [30-33]. T regulatory cells play a central role in the mechanism of effective SIT with an important effect on the down regulation of the Th2 response [34]. One mechanism by which immunotherapy suppresses the allergic response is through an increase in the production of specific IgG antibodies, primarily of the IgG4 subtype. In the context of IgE-mediated allergy, the appearance of IgG4 antibodies is usually associated with a decrease in symptoms. This is likely to be due, at least in part, to an allergen-blocking effect at the mast cell level and/or at the level of the antigen-presenting cell (preventing IgE-facilitated activation of T cells) [35]. Because the production of IgE against normally harmless antigens is the cause of the allergic responses, the production of antigen specific IgG antibodies, can antagonise and block the allergic inflammatory cascade resulting from antigen recognition by IgE. Therefore, the shift in balance between IgE and IgG4 is essential to successful allergen SIT. It has been demonstrated that allergen-specific IgG4 antibodies with potent inhibitory activity against IgE persist after treatment discontinuation and could account for long-term clinical tolerance [36]. Induction of specific IgG4 has the capacity to influence the allergic response in different ways (Figure 1), and is related to immunological effector mechanisms, also responsible for the reduced late-phase hyperreactivity and ongoing allergic inflammation. Details are described in Textbox 1.

Studies have shown that symptomatic improvement correlates with reductions in eosinophils and IL-5 expression in the nasal mucosa [32] during the pollen season, as well as with increased interferon-gamma (INF) production [37]. Immunotherapy induces an IL-12-response shown to be inversely related to IL-4 production and to promote a Th1-response estimated as interferon-gamma production which promotes B-cells into IgG production [31]. Several studies have shown that immunotherapy induces a new regulatory T-cell response characterised by induction of IL-10 [38,39] which precedes the inhibitory IgG4 antibody activity [40]. TGF-β is also a T-regulatory cell mediator induced by immunotherapy [41] which is responsible for the down regulation of the Th2 response - reducing IL-5 production [33] and preventing allergen-exposure-induced eosinophilia and inflammation [32]. The importance of T regulatory cells in allergy and in relation to immunotherapy has been carefully reviewed [34,42,43]. The left side of Figure 1 summarises basic immunological changes during immunotherapy. The immunological mechanisms described here illustrate the close connection between the basic immunological response to immunotherapy and the humoral response, characterised by the induction of competitive specific antibodies accompanying and potentially leading to clinical benefits.

### Textbox 1

Different IgG4 related immunological effects acting as a competitive response to that of specific IgE, result in clinical effects as reduction in symptoms and the need for medication, and a reduced inflammatory response to allergen presentation (Figure 1).

#### Inhibition of mediator release

• Inhibition of mediator release could be caused by direct inhibition of the allergen-IgE interaction or through co-aggregation of the inhibitory FcγRIIB and FcγRI [44]

• SIT-induced IgG inhibited histamine release from basophils [45]

#### Blocking antibody

• Specific IgG antibodies acts by a "dilution" of the antigen exposure to specific IgE antibodies by binding to epitopes on the surface of the allergen, and thereby directly reducing the antigen presenting capacity by the relevant cell types [46]

#### Facilitated Antigen Presentation (FAP)

• IgE-facilitated antigen presentation results in the activation and subsequent proliferation of allergen-specific T-cells at extremely low concentrations of allergen and activates Th2 cells which then produce important cytokines (IL-4, IL-13) inducing further IgE synthesis [47]

• Immunotherapy-induced specific IgG antibodies can also affect and inhibit IgE- facilitated antigen presentation (FAP) and activation of Th2-cells, and thereby significantly reduce the allergic response [23,45,48]

There is a time dependence in serum inhibitory activity of Facilitated Antigen Binding which seem to be consistent with the continued increase in clinical benefits following prolonged treatment with immunotherapy [49]

### Clinical effectiveness of SIT

The first priority for respiratory allergic patients is usually the treatment of allergic symptoms as these appear as a consequence of allergen exposure. Different symptomatic drugs have the capacity to reduce the symptoms either on a rescue basis or when taken daily [4,22]. From a regulatory point of view the primary focus on the benefit from anti-allergic treatments is usually on the potential reduction in symptoms. Together with the potential range of benefits following SIT, reduction in symptoms is mandatory. Recently, an overview of how effective SIT is in reducing symptoms including a comparison to the effectiveness of symptomatic treatment options was published [50]. The data support that already during the first seasonal exposure after initiation of SCIT a reduction in symptoms at least as potent as the reduction following treatment with symptomatic drugs can be achieved.

Together with the significant reduction in allergic symptoms, several studies on SIT have documented a significant reduction in the need for symptomatic drugs on short term as well as during long-term treatment [51-54] even in patients with severe allergic rhinoconjunctivitis, who fail to respond adequately to symptomatic treatment [55]. The reduction in symptomatic medication achieved early after initiation of treatment with SIT has been shown for pollens [52] as well as perennial allergens [56]. Also, the long-term benefit even after termination of SIT has been shown [57,58]. The most comprehensive overview of the outcome on reduction of the need for symptomatic medication is provided by the recent Cochrane reviews covering SIT studies with subcutaneous and sublingual application of SIT for rhinoconjunctivitis as well as asthma [59-61]. If patients were sensitised to other allergens, this did not influence the response to the treatment for their grass allergy [55], suggesting that SIT can be efficiently used even in polysensitised patients. Both adults and children with house dust mite-induced allergic asthma, benefit from SIT which results in a steroid-sparing effect by following guideline-defined asthma control [62,63]. This is of particular importance as allergic asthma is frequently associated with rhinitis [64] with the consequent need of simultaneous nasal steroid therapy as well as some concern arising from possible adverse effects of long-term treatment with inhaled steroids in children [65].

### Endpoints and combined symptom and medication score in SIT trials

The World Allergy Organization has proposed a model for the standardisation of clinical trials of allergen specific immunotherapy for respiratory allergic symptoms [21]. They suggest defining the primary outcome of immunotherapy trials as the total clinical score, calculated as the sum of symptom score plus drugs usage calculated as one score. The European Medicines Agency (EMA guideline "Guideline on the clinical development of products for specific immunotherapy for the treatment of allergic diseases") has also described the issues that the use of rescue medication has a direct impact on symptom severity [18]. An estimate of the power as well as the number of subjects to be included is mandatory to the outcome of clinical studies and the primary endpoint of a clinical study should be the variable capable of providing the most clinically relevant and convincing evidence directly related to the primary objective of the trial. It is also mandatory in confirmative clinical trials to define the endpoint up front. It may sometimes be desirable to use more than one primary variable, each of which could be sufficient to cover the range of effects of the therapies. For clinical trials on allergen specific immunotherapy this could be the use of symptom score as well as medication score reduction [66]. The extent of inter correlation among the proposed primary variables may be considered.

Different methods for combining symptom score and intake of rescue medication have been described [66,67] but there is no guideline for how this combined score should be established, and from case to case the method used must be pre-specified and justified. In order to further develop the methods for one and only one final endpoint for trials on SIT, new methods to establish a clinical relevant score evaluating the potential reduction in allergic symptoms in combination with the potential reduced need for symptomatic medication have been assessed [51,68,69]. Recently, the first validation of a combined symptom/medication score strategy was published as the "Allergy-Control-SCORE™". Based on a comparison of the symptoms and need for medication during the pollen season between allergic subjects and healthy controls, they proved a reliable instrument in distinguishing between allergic patients and controls, which is also able to access the severity of disease [70]. After the final endpoint has been defined and the results obtained, it is possible to look for secondary parameters and explorative outcomes which are extremely useful for the establishment of new hypotheses and future research [66,71].

### Clinical effect levels

In this paper the effects of immunotherapy are categorised into the following levels of benefit whose immunological characteristics are explained above.

• Early effect - reduction in symptoms/need for medication

• Progressive effect - further reduction in symptoms/need for medication and reduction in hyperresponsiveness/late-phase response

• Persistent effect - long-term reduced symptoms/need for medication long-term reduced hyperresponsiveness/late phase response after end of treatment period

• Preventive effect - prevention of new sensitivities and progression of disease (rhinitis into asthma)

• Immunological effect - immune modulation and tolerance - immunological changes that lead to a potentially curative effect

These correspond to the different levels of clinical effects which can be claimed for the registration of products for SIT as defined by the EMA [18] (Textbox 2). Evidence for the different claims is summarised below.

### Textbox 2

Depending on the clinical documentation available and study duration different claims for efficacy are possible:

• Treatment of allergic symptoms: Short term clinical trials conducted to show efficacy in the first pollen season after commencing specific immunotherapy or to show efficacy in perennial allergies after some months of treatment

• Sustained clinical effect: Maintenance of significant and clinically relevant efficacy over two to three treatment years

• Long-term efficacy and disease modifying effect: Sustained significant and clinically relevant efficacy in post-treatment years

• Curing allergy: Sustained absence of allergic symptoms in post-treatment years

### Early effect -reduction in symptoms/need for medication

It is evident that very early on and during the first seasonal exposure after initiation of immunotherapy -or alternatively when patients have reached the top or maintenance dosage [30,55,72,73], they experience an improvement in allergy symptoms (rhinoconjunctivitis and/or asthmatic symptoms) and a need for rescue medication. The early effect of SIT on rhinoconjunctivitis symptom scores has been confirmed in the so-called "big trials" which recruited hundreds of grass pollen allergic patients [55,74,75]. In sublingual tablet studies the positive effects of at least 16 weeks of treatment have been documented [74,75] and a single study using an adjuvant (MPL) in combination with a grass allergen extract also indicate efficacy after only 4 weeks with 4 injections [76,77].

Pre-seasonal short-term therapy normally including six weeks of treatment has been shown to be clinically effective as early as in the first season [51,78-80]. A six-month treatment period with recombinant grass pollen major allergens has also proved effective [81]. A combined symptom/medication score strategy has recently been used and confirmed the clinical efficacy for short term injection therapy [51,68], as well as for sublingual tablet treatment [66].

### Progressive effect - further reduction in symptoms/need for medication and reduction in hyperresponsiveness/late-phase response

During a long-term treatment period of up to four years, persistent effects and further increased clinical and immunological benefits are achieved. This has been shown for grass, cat and dog, and house dust mites [28,82,83]. In a study using two consecutive pre-seasonal short-courses of a grass pollen allergoid, using a combined symptom medication score it was clear that although the treatment was clinically effective in the first season, its clinical efficacy was even better in the second seasonal [51]. An open continuation of this study including a third pre-seasonal treatment period confirmed the increased efficacy potential which prolonged immunotherapy treatment provides [54]. When continuing the treatment for more than 12 months it is possible to introduce non-specific efficacy parameters, seen as a decrease in the patient's non-specific bronchial hyper responsiveness as an indicator of inflammation. This was shown in patients suffering from house dust mite related allergic asthma, who increased their tolerance to bronchial challenge with house dust mite allergen after six months, but it was only after 18 months of SIT that a persistent reduced bronchial inflammation and hyperresponsiveness measured by bronchial challenge with metacholine was found [84]. It has also been observed that two years of immunotherapy prevented the seasonal onset of bronchial hyperresponsiveness in grass pollen allergic patients with rhinitis and asthma [29]. According to a meta-analysis on asthma, SCIT significantly reduced allergen specific bronchial hyperresponsiveness, which was evaluated in the majority of the studies done after one year of treatment [61]. The finding that SIT is able to improve specific bronchial hyperreactivity is important from a clinical point of view. Indeed, patients with brittle allergic asthma are at risk of sudden deterioration when exposed to increased levels of an aeroallergen to which they are sensitive, as in the case of mould allergic patients. Currently, the measurement of BHR is the only accurate method of assessing such a risk and any intervention which reduces the risk of an acute episode of asthma under these circumstances would be clinically useful. Moreover, a high percentage of children and adults also develop a late asthmatic response after an allergen specific bronchial challenge [85], the presence of which is considered an experimental model close to resembling chronic bronchial allergic inflammation. Subcutaneous SIT is able to decrease not only early, but also late asthmatic responses following allergen specific bronchial challenge, thus confirming the anti-inflammatory effect of the treatment in the lung [53].

A Th2/Th1 response towards normal balance is usually associated with a positive clinical effect of immunotherapy. In grass pollen allergic patients undergoing injection immunotherapy it has been shown that a significant change towards a Th1 response measured by the level of interferon-gamma in peripheral blood was achieved after a 12-month treatment period, but not after just three months treatment -despite a well-documented symptomatic effect being found after three months [30]. Administering a short four-week therapy with a grass allergen extract together with an MPL adjuvant showed that specific IgG and IgG4 levels increased only slightly in year one of SIT, but which became highly significant in year two [86]. A progressive immunological change as indicated by the significant induction of non-IgE competitive antibodies was also observed in year two of treatment with a sublingual tablet for grass pollen allergy whose clinical efficacy comparable to the benefits of year one [87]. In a three-year long-term SIT study in grass allergic patients with both rhinoconjunctivitis and asthma the asthma symptoms had completely cleared after 3 years with a general progression in clinical effect over the years. Symptoms and the need for medication was generally reduced from the beginning although nasal symptoms were not reduced during the first season - again indicating that continuing the treatment will increase the benefits [88].

### Persistent effect -long-term reduced symptoms/need for medication -long-term reduced hyperresponsiveness/late-phase response after end of treatment period

Recently, wider studies have confirmed the long-term clinical potential of SIT after termination of treatment. In the Preventive Allergy Treatment Study (PAT) children treated for 3 years with pollen immunotherapy (grass and/or birch) showed a consistent reduction in their symptom score rated by Visual Analogue Score (VAS) and by objective measures of conjunctival sensitivity (CPT). The children were tested respectively two and seven years after discontinuation of treatment [89,90]. The sustained long-term and disease-modifying effects of immunotherapy have also been confirmed in a large-scale randomised, double-blind, placebo-controlled sublingual trial with a two-year follow-up after three consecutive years of treatment with a sublingual grass allergen tablet [58,91]. The key study which confirms the long-term persistent clinical and also immunological effect measured on late-phase skin response to an allergen, is the double blind randomised study where patients treated for grass pollen allergy with SCIT over a three-to-four year span were followed up to three years after cessation of therapy and compared to a SCIT naive matched group of patients [57]. Other studies have shown up to eight years persistent long-term clinical effects after termination of two to three years of SIT for grass pollen, tree pollen, animal hair and dander and house dust mites [92-96]. In cat allergic patients with mild to moderate asthma not only was the reactivity to cat allergen reduced following immunotherapy, but also a reduction in specific as well as non-specific hyperresponsiveness occurred during the five-year follow up period [94]. In a small open controlled three year consecutive pre-seasonal treatment study with an allergoid persistent clinical efficacy in children for as long as up to 12 years after discontinuation of the treatment was shown [97,98].

### Preventive effect -prevention of new sensitivities and progression of disease (rhinitis into asthma)

#### Evidence for prevention of asthma - the PAT study and background

The Preventive Allergy Treatment study (PAT) [9] was the first large prospective randomised controlled long-term follow-up study designed to show whether SIT can prevent the development of asthma in children suffering from seasonal allergic rhinoconjunctivitis caused by allergy to birch and/or grass pollen. SIT was administered for three years, after which the children were evaluated for the development of asthma. Metacholine bronchial provocation tests were carried out during the relevant season(s) and during winter. Unexpectedly, as a consequence of the careful study examination at baseline, before commencing SIT, it was found that 20% of the children had undiscovered mild asthmatic symptoms during the pollen season(s), and that more than a third had significant seasonal ongoing bronchial hyperresponsiveness as revealed by metacholine challenge. Among those without asthma at baseline, the group treated with SIT showed a significant positive odds ratio for reducing the risk for development of asthma (odds ratio 2.52; p < 0.001). Another study using sublingual immunotherapy with grass allergen extract for 3 consecutive years also showed potential for the prevention of developing new asthma in children during the treatment period [99]. In this open randomised trial in children aged 5-14 years, patients were treated with a standardised mixture of 5 grasses. The PAT study initiative was based on a list of clinical studies which had all shown preventive potential. Among these, the classic 14-year long-term follow up study in children by Johnstone and Dutton who investigated SIT for the prevention of exacerbation and development of asthma. A highly significant reduction in the number of patients with asthma was reported at the time of follow-up which corresponded to the date of the children's 16^th ^birthday. Only 22% of the placebo treated children were free of asthma compared to 72% of the SIT treated children. The children were initially treated for four years with individual mixtures of allergens and the clinical effect as well as the potential prevention of asthmatic symptoms was dose related - the strongest being in children who received the highest doses of allergen [100]. In a two-year placebo controlled study on the effect of immunotherapy in patients with allergic rhinoconjunctivitis caused by house dust mite allergy, the authors had selected children and adults with coexisting BHR. Besides the finding that immunotherapy reduced the provocative dose of metacholine 4-fold, they also reported that none of the SIT treated patients had developed symptoms of asthma during the 2 year study period [101].

#### Evidence for the prevention of new allergies

An early study by Johnstone showed that SCIT could reduce the risk of development of new allergic sensitisations as they found that no children during a 4 year course of high dose immunotherapy developed new IgE sensitisations compared to 25% of those in the control group [102]. In the last two decades several studies using SIT have confirmed such findings. Two studies have shown the reduction in new sensitisations in children who were treated for house dust mite allergy.

In one study, monosensitised children treated for three years with SIT compared to non-treated controls, showed a significant reduction in the development of new allergic sensitivities (45% developed no new sensitisations at all) although none of the control patients remained free of the development of one or more new sensitisations [103]. In the other study measuring sensitisations to inhalant allergens three years after termination of house dust mite immunotherapy, two thirds of the control group children had developed one or more new sensitisations revealed by skin prick testing and specific IgE compared to only a quarter with new sensitivities in the SIT group [104].

A very large follow-up study including more than 7182 monosensitised patients with different allergies and with allergic rhinitis and/or asthma were treated with SCIT for four years and compared to 1214 open controls treated only with symptomatic drugs. Sixty-eight percent of controls had developed one or more new sensitisations compared to 27% in immunotherapy treated patients and at a follow-up visit three years after termination of the treatment period, 75% of controls had developed new sensitizations compared to 25% in the active group [105]. In a group of children with grass allergy followed up for 12 years the data revealed fewer new allergies in the active group treated with a consecutive three year pre-seasonal short-term treatment compared with controls [98,99]. Most studies on prevention of new sensitizations have been performed using SCIT, but SLIT has also shown its potential for reducing the risk for new allergies from 38% of control group patients to 5.9% of actively treated patients [106].

#### Evidence for long term prevention

The long term asthma preventive potential of SIT was first described in a tree pollen immunotherapy study in which 36 adult patients received immunotherapy. During the long-term six year follow-up period after the termination of treatment with standardised tree pollen allergen extracts for two years, none of the patients initially suffering from rhinitis alone had developed asthma during the eight-year study period [94]. The five and ten year follow-up of the PAT study are the first prospective follow-up studies testing whether SIT can prevent the long-term development of asthma and whether the clinical effects persist in children suffering from seasonal allergic rhinoconjunctivitis caused by allergy to birch and/or grass pollen as these children mature. After the total SIT period of three years, the children were evaluated for development of asthma and then re-evaluated after a total of five years. The evaluation showed that immunotherapy reduces progression from allergic rhinoconjunctivitis to asthma after three years of SIT [9] and at the five year follow-up two years after SIT termination [89]. The actively treated children had persistently significantly less asthma at the five year follow-up (odds ratio 3.1; *p *< 0.01). The significant improvements in allergic rhinoconjunctivitis symptom and medications scores as well as in the conjunctival sensitivity to birch and grass observed to persist at five-year follow-up, also persisted at the ten-year follow-up. As at the five-year follow-up, fewer actively treated subjects had developed asthma at the ten-year follow-up (odds ratio 2.5 (1.1-5.9) [90]. After the complete study, the longitudinal treatment effect including all observations at three-, five- and ten-year follow-up was highly statistically significant (*p *= 0.0075) and the odds ratio for not developing asthma was 4.6 (95% CI (1.5; 13.7)) in favour of SIT over the ten-year period. The study also showed that bronchial hyperresponsiveness at baseline was associated with an increased risk of later development of asthma (*p *= 0.002) and in children with seasonal allergic rhinitis evaluation of BHR should be recommended in the indication for immunotherapy.

#### Only SIT has a long term and preventive potential

Neither symptomatic treatment nor different avoidance measures for allergy have so far documented the long-term or preventive benefits. Various strategies for the prevention of the development of allergic rhinoconjunctivitis and asthma have been proposed including allergen avoidance, pharmacological treatment (antihistamines and steroids) and SIT. Allergen avoidance is hardly applicable for many allergen sources and only a limited reduction in exposure can be achieved by modification of life habits (Prevention of Allergy and Allergic Asthma -WHO/WAO Geneva, 2002) [107]. Secondary prevention addressing children suffering from atopic dermatitis to prevent respiratory symptoms and further disease progression into asthma involves traditional pharmacotherapy with antihistamines. While treatment with antihistamines does provide symptomatic relief, it does not modify long term outcomes in children as the natural course of the disease is not altered. A recent large multi-centre trial in which children with atopic dermatitis were given cetirizine, failed to reduce the development of asthma [108].

A potential tertiary preventive measure for worsening of asthma through early treatment with inhaled steroids in children with episodic wheezing has been suggested, but recent studies on the capacity of inhaled steroid therapy during early symptomatic episodes of wheezing to delay progression to persistent disease have failed to show any preventive potential [109,110].

## Conclusion

Allergen specific immunotherapy is the only treatment that interferes with the basic pathophysiological mechanisms of the allergic disease and thereby carries the potential for changes in the long-term prognosis of respiratory allergy. SIT should be recognised not only as first-line therapeutic treatment for allergic rhinoconjunctivitis but also as secondary preventive treatment for respiratory allergic diseases. Together with the long term clinical experience available, SIT is an important treatment for prevention of asthma in patients with allergic rhinitis and it makes asthma control easier to accomplish. Immunotherapy seems to reduce the development of new allergic sensitivities as measured by skin prick test as well as specific IgE measurements, and long term follow-up on immunotherapy studies demonstrate that specific immunotherapy with modern pharmaceutically standardised allergen extracts shows persistent long term effect on clinical symptoms after termination of treatment and long-term, preventive effect on later development of asthma in children with seasonal rhinoconjunctivitis. These are important added benefits to the immediate clinical effect which is comparable to other treatment options and has shown to progressively improve during treatment periods. It is so far the only treatment for allergic diseases which has shown to be able to prevent worsening of disease and development of asthma. In these years, a number of large ongoing clinical studies with the primary purpose of establishing the EMA guideline documentation needed for registration of pharmaceutical products will certainly add further to our basic knowledge about the potential immunological modifications and long-term options. This is of great importance for the optimization of the future treatment of the increasing number of patients suffering from respiratory allergic diseases.

## Abbreviations

SIT: Allergen Specific Immunotherapy; SCIT: Subcutaneous Immunotherapy; SLIT: Sublingual Immunotherapy; BHR: Bronchial Hyperresponsiveness; CPT: Conjunctival Provocation Test; VAS: Visual Analogue Scale.

## Competing interests

The authors declare that they have no competing interests.

## Authors' contributions

LJ wrote the manuscript. MB revised and added to the manuscript. UW revised the manuscript. All authors read and approved the final manuscript.

## References

[B1] Braunstahl GJ Prins JB Kleinjan A Overbeek SE Hoogsteden HC Fokkens WJ Nose and lung cross-talk in allergic airways disease Clin Exp All Rev 2003 3 38 42 10.1046/j.1472-9725.2003.00060.x

[B2] Togias A Mechanisms of nose-lung interaction Allergy 1999 54 Suppl 57 94 105 10565484 10.1111/j.1398-9995.1999.tb04410.x

[B3] Cruz AA Popov T Pawankar R Annesi-Maesano I Fokkens W Kemp J Ohta K Price D Bousquet J Common characteristics of upper and lower airways in rhinitis and asthma: ARIA update, in collaboration with GA(2)LEN Allergy 2007 62 Suppl 84 1 41 17924930 10.1111/j.1398-9995.2007.01551.x

[B4] Bousquet J Khaltaev N Cruz AA Denburg J Fokkens WJ Togias A Zuberbier T Baena-Cagnani CE Canonica GW van Weel C Allergic Rhinitis and its Impact on Asthma (ARIA) 2008 update (in collaboration with the World Health Organization, GA(2)LEN and AllerGen) Allergy 2008 63 Suppl 86 8 160 18331513 10.1111/j.1398-9995.2007.01620.x

[B5] Ruokonen M Kaila M Haataja R Korppi M Paassilta M Allergic rhinitis in school-aged children with asthma - still under-diagnosed and under-treated? A retrospective study in a children's hospital Pediatr Allergy Immunol 2010 21 e149 e154 10.1111/j.1399-3038.2009.00891.x 19594853

[B6] Bousquet J Gaugris S Kocevar VS Zhang Q Yin DD Polos PG Bjermer L Increased risk of asthma attacks and emergency visits among asthma patients with allergic rhinitis: a subgroup analysis of the investigation of montelukast as a partner agent for complementary therapy [corrected] Clin Exp Allergy 2005 35 723 727 10.1111/j.1365-2222.2005.02251.x 15969661

[B7] Linneberg A Henrik NN Frolund L Madsen F Dirksen A Jorgensen T The link between allergic rhinitis and allergic asthma: a prospective population-based study The Copenhagen Allergy Study Allergy 2002 57 1048 1052 10.1034/j.1398-9995.2002.23664.x 12359002

[B8] Settipane RJ Hagy GW Settipane GA Long-term risk factors for developing asthma and allergic rhinitis: a 23-year follow-up study of college students Allergy Proc 1994 15 21 25 10.2500/108854194778816634 8005452

[B9] Moller C Dreborg S Ferdousi HA Halken S Host A Jacobsen L Koivikko A Koller DY Niggemann B Norberg LA Pollen immunotherapy reduces the development of asthma in children with seasonal rhinoconjunctivitis (the PAT-study) J Allergy Clin Immunol 2002 109 251 256 10.1067/mai.2002.121317 11842293

[B10] Ciprandi G Buscaglia S Pesce G Pronzato C Ricca V Parmiani S Bagnasco M Canonica GW Minimal persistent inflammation is present at mucosal level in patients with asymptomatic rhinitis and mite allergy J Allergy Clin Immunol 1995 96 971 979 10.1016/S0091-6749(95)70235-0 8543756

[B11] Silvestri M Rossi GA Cozzani S Pulvirenti G Fasce L Age-dependent tendency to become sensitized to other classes of aeroallergens in atopic asthmatic children Ann Allergy Asthma Immunol 1999 83 335 340 10.1016/S1081-1206(10)62674-9 10541426

[B12] Linneberg A Nielsen NH Madsen F Frolund L Dirksen A Jorgensen T Pets in the home and the development of pet allergy in adulthood The Copenhagen Allergy Study Allergy 2003 58 21 26 10.1034/j.1398-9995.2003.23639.x 12580802

[B13] Cox L Wallace D Specific allergy immunotherapy for allergic rhinitis: subcutaneous and sublingual Immunol Allergy Clin North Am 2011 31 561 599 10.1016/j.iac.2011.05.001 21737043

[B14] Jacobsen L Preventive aspects of immunotherapy: prevention for children at risk of developing asthma Ann Allergy Asthma Immunol 2001 87 43 46 11476475 10.1016/s1081-1206(10)62194-1

[B15] Noon L Prophylactic inoculation against hay fever Lancet 1911 177 1572 1573 10.1016/S0140-6736(00)78276-6

[B16] Larche M Akdis CA Valenta R Immunological mechanisms of allergen-specific immunotherapy Nat Rev Immunol 2006 6 761 771 10.1038/nri1934 16998509

[B17] Casale TB Stokes JR Future forms of immunotherapy J Allergy Clin Immunol 2011 127 8 15 10.1016/j.jaci.2010.10.034 21094518

[B18] Committee for Medicinal Products for Human Use Guideline on the Clinical Development of Products for Specific Immunotherapy for the Treatment of Allergic Diseases European Medicines Agency 2008 http://www.ema.europa.eu/docs/en_GB/document_library/Scientific_guideline/2009/09/WC500003605.pdf

[B19] Committee for Medicinal Products for Human Use Guideline on Allergen Products: Production and Quality Issues European Medicines Agency 2008 http://www.ema.europa.eu/docs/en_GB/document_library/Scientific_guideline/2009/09/WC500003333.pdf

[B20] Bousquet J Van Cauwenberge P Khaltaev N Allergic rhinitis and its impact on asthma J Allergy Clin Immunol 2001 108 S147 S334 10.1067/mai.2001.118891 11707753

[B21] Canonica GW Baena-Cagnani CE Bousquet J Bousquet PJ Lockey RF Malling HJ Passalacqua G Potter P Valovirta E Recommendations for standardization of clinical trials with Allergen Specific Immunotherapy for respiratory allergy. A statement of a World Allergy Organization (WAO) taskforce Allergy 2007 62 317 324 10.1111/j.1398-9995.2006.01312.x 17298350

[B22] Batemann ED GINA Report, Global Strategy for Asthma Management and Prevention GINA No 95-3659 2009 National Heart, Lung and Blood Institute, National Institute of Health 1 92

[B23] James LK Durham SR Update on mechanisms of allergen injection immunotherapy Clin Exp Allergy 2008 38 1074 1088 10.1111/j.1365-2222.2008.02976.x 18691292

[B24] Novak N Bieber T Allam JP Immunological mechanisms of sublingual allergen-specific immunotherapy Allergy 2011 66 733 739 10.1111/j.1398-9995.2010.02535.x 21251016

[B25] Shamji MH Durham SR Mechanisms of immunotherapy to aeroallergens Clin Exp Allergy 2011 41 1235 1246 10.1111/j.1365-2222.2011.03804.x 21762223

[B26] Passalacqua G Durham SR Allergic rhinitis and its impact on asthma update: allergen immunotherapy J Allergy Clin Immunol 2007 119 881 891 10.1016/j.jaci.2007.01.045 17418661

[B27] Rak S Lowhagen O Venge P The effect of immunotherapy on bronchial hyperresponsiveness and eosinophil cationic protein in pollen-allergic patients J Allergy Clin Immunol 1988 82 470 480 10.1016/0091-6749(88)90021-8 3170995

[B28] Walker SM Varney VA Gaga M Jacobson MR Durham SR Grass pollen immunotherapy: efficacy and safety during a 4-year follow-up study Allergy 1995 50 405 413 10.1111/j.1398-9995.1995.tb01170.x 7573829

[B29] Walker SM Pajno GB Lima MT Wilson DR Durham SR Grass pollen immunotherapy for seasonal rhinitis and asthma: a randomized, controlled trial J Allergy Clin Immunol 2001 107 87 93 10.1067/mai.2001.112027 11149996

[B30] Ebner C Siemann U Bohle B Willheim M Wiedermann U Schenk S Klotz F Ebner H Kraft D Scheiner O Immunological changes during specific immunotherapy of grass pollen allergy: reduced lymphoproliferative responses to allergen and shift from TH2 to TH1 in T-cell clones specific for Phl p 1, a major grass pollen allergen [see comments] Clin Exp Allergy 1997 27 1007 1015 10.1111/j.1365-2222.1997.tb01252.x 9678832

[B31] Hamid QA Schotman E Jacobson MR Walker SM Durham SR Increases in IL-12 messenger RNA + cells accompany inhibition of allergen-induced late skin responses after successful grass pollen immunotherapy [see comments] J Allergy Clin Immunol 1997 99 254 260 10.1016/S0091-6749(97)70106-4 9042055

[B32] Wilson DR Nouri-Aria KT Walker SM Pajno GB O'Brien F Jacobson MR Mackay IS Durham SR Grass pollen immunotherapy: symptomatic improvement correlates with reductions in eosinophils and IL-5 mRNA expression in the nasal mucosa during the pollen season J Allergy Clin Immunol 2001 107 971 976 10.1067/mai.2001.115483 11398073

[B33] Wachholz PA Nouri-Aria KT Wilson DR Walker SM Verhoef A Till SJ Durham SR Grass pollen immunotherapy for hayfever is associated with increases in local nasal but not peripheral Th1: Th2 cytokine ratios Immunology 2002 105 56 62 10.1046/j.1365-2567.2002.01338.x 11849315 PMC1782637

[B34] Ozdemir C Kucuksezer UC Akdis M Akdis CA Specific immunotherapy and turning off the T cell: how does it work? Ann Allergy Asthma Immunol 2011 107 381 392 10.1016/j.anai.2011.05.017 22018608

[B35] Aalberse RC Stapel SO Schuurman J Rispens T Immunoglobulin G4: an odd antibody Clin Exp Allergy 2009 39 469 477 10.1111/j.1365-2222.2009.03207.x 19222496

[B36] James LK Shamji MH Walker SM Wilson DR Wachholz PA Francis JN Jacobson MR Kimber I Till SJ Durham SR Long-term tolerance after allergen immunotherapy is accompanied by selective persistence of blocking antibodies J Allergy Clin Immunol 2011 127 509 516 10.1016/j.jaci.2010.12.1080 21281875

[B37] Durham SR Ying S Varney VA Jacobson MR Sudderick RM Mackay IS Kay AB Hamid QA Grass pollen immunotherapy inhibits allergen-induced infiltration of CD4+ T lymphocytes and eosinophils in the nasal mucosa and increases the number of cells expressing messenger RNA for interferon-gamma J Allergy Clin Immunol 1996 97 1356 1365 10.1016/S0091-6749(96)70205-1 8648033

[B38] Savolainen J Jacobsen L Valovirta E Sublingual immunotherapy in children modulates allergen-induced in vitro expression of cytokine mRNA in PBMC Allergy 2006 61 1184 1190 10.1111/j.1398-9995.2006.01206.x 16942566

[B39] Nouri-Aria KT Wachholz PA Francis JN Jacobson MR Walker SM Wilcock LK Staple SQ Aalberse RC Till SJ Durham SR Grass pollen immunotherapy induces mucosal and peripheral IL-10 responses and blocking IgG activity J Immunol 2004 172 3252 3259 14978133 10.4049/jimmunol.172.5.3252

[B40] Francis JN James LK Paraskevopoulos G Wong C Calderon MA Durham SR Till SJ Grass pollen immunotherapy: IL-10 induction and suppression of late responses precedes IgG4 inhibitory antibody activity J Allergy Clin Immunol 2008 121 1120 1125 10.1016/j.jaci.2008.01.072 18374405

[B41] Jutel M Akdis M Budak F Aebischer-Casaulta C Wrzyszcz M Blaser K Akdis CA IL-10 and TGF-beta cooperate in the regulatory T cell response to mucosal allergens in normal immunity and specific immunotherapy Eur J Immunol 2003 33 1205 1214 10.1002/eji.200322919 12731045

[B42] Robinson DS Larché M Durham S Tregs and allergic disease J Clin Invest 2004 114 1389 1397 15545986 10.1172/JCI23595 PMC525754

[B43] Akdis CA Akdis M Mechanisms of allergen-specific immunotherapy J Allergy Clin Immunol 2011 127 18 27 10.1016/j.jaci.2010.11.030 21211639

[B44] Kepley CL Cambier JC Morel PA Lujan D Ortega E Wilson BS Oliver JM Negative regulation of FcepsilonRI signaling by FcgammaRII costimulation in human blood basophils J Allergy Clin Immunol 2000 106 337 348 10.1067/mai.2000.107931 10932079

[B45] Wurtzen PA Lund G Lund K Arvidsson M Rak S Ipsen H A double-blind placebo-controlled birch allergy vaccination study II: correlation between inhibition of IgE binding, histamine release and facilitated allergen presentation Clin Exp Allergy 2008 38 8 1290 1301 10.1111/j.1365-2222.2008.03020.x 18510696

[B46] Wachholz PA Soni NK Till SJ Durham SR Inhibition of allergen-IgE binding to B cells by IgG antibodies after grass pollen immunotherapy J Allergy Clin Immunol 2003 112 915 922 10.1016/S0091-6749(03)02022-0 14610480

[B47] van Neerven RJ Knol EF Ejrnaes A Wurtzen PA IgE-mediated allergen presentation and blocking antibodies: regulation of T-cell activation in allergy Int Arch Allergy Immunol 2006 141 119 129 10.1159/000094714 16864979

[B48] van Neerven RJ Wikborg T Lund G Jacobsen B Brinch-Nielsen A Arnved J Ipsen H Blocking antibodies induced by specific allergy vaccination prevent the activation of CD4+ T cells by inhibiting serum-IgE-facilitated allergen presentation J Immunol 1999 163 2944 2952 10453043

[B49] Scadding GW Shamji MH Jacobson MR Lee DI Wilson D Lima MT Pitkin L Pilette C Nouri-Aria K Durham SR Sublingual grass pollen immunotherapy is associated with increases in sublingual Foxp3-expressing cells and elevated allergen-specific immunoglobulin G4, immunoglobulin A and serum inhibitory activity for immunoglobulin E-facilitated allergen binding to B cells Clin Exp Allergy 2010 40 598 606 20184605 10.1111/j.1365-2222.2010.03462.x

[B50] Matricardi PM Kuna P Panetta V Wahn U Narkus A Subcutaneous immunotherapy and pharmacotherapy in seasonal allergic rhinitis: A comparison based on meta-analyses J Allergy Clin Immunol 2011 128 791 799 10.1016/j.jaci.2011.03.049 21620452

[B51] Corrigan CJ Kettner J Doemer C Cromwell O Narkus A Efficacy and safety of preseasonal-specific immunotherapy with an aluminium-adsorbed six-grass pollen allergoid Allergy 2005 60 801 807 10.1111/j.1398-9995.2005.00790.x 15876311

[B52] Bodtger U Poulsen LK Jacobi HH Malling HJ The safety and efficacy of subcutaneous birch pollen immunotherapy - a one-year, randomised, double-blind, placebo-controlled study Allergy 2002 57 297 305 10.1034/j.1398-9995.2002.1o3532.x 11906359

[B53] Arvidsson MB Lowhagen O Rak S Allergen specific immunotherapy attenuates early and late phase reactions in lower airways of birch pollen asthmatic patients: a double blind placebo-controlled study Allergy 2004 59 74 80 14674937 10.1046/j.1398-9995.2003.00334.x

[B54] Williams A Henzgen M Rajakulasingam K Additional benefit of a third year of specific grass pollen allergoid immunotherapy in patients with seasonal allergic rhinitis Eur Ann Allergy Clin Immunol 2007 39 123 126 17523386

[B55] Frew AJ Powell RJ Corrigan CJ Durham SR Efficacy and safety of specific immunotherapy with SQ allergen extract in treatment-resistant seasonal allergic rhinoconjunctivitis J Allergy Clin Immunol 2006 117 319 325 10.1016/j.jaci.2005.11.014 16461133

[B56] Garcia-Robaina JC de la TF Sanchez I Fernandez-Caldas E Casanovas M Successful management of mite-allergic asthma with modified extracts of Dermatophagoides pteronyssinus and Dermatophagoides farinae in a double-blind, placebo-controlled study J Allergy Clin Immunol 2006 118 1026 1032 10.1016/j.jaci.2006.07.043 17088125

[B57] Durham SR Walker SM Varga EM Jacobson MR O'Brien F Noble W Till SJ Hamid QA Nouri-Aria KT Long-term clinical efficacy of grass-pollen immunotherapy [see comments] N Engl J Med 1999 341 468 475 10.1056/NEJM199908123410702 10441602

[B58] Durham SR Emminger W Kapp A Colombo G de Monchy JG Rak S Scadding GK Andersen JS Riis B Dahl R Long-term clinical efficacy in grass pollen-induced rhinoconjunctivitis after treatment with SQ-standardized grass allergy immunotherapy tablet J Allergy Clin Immunol 2010 125 131 138 10.1016/j.jaci.2009.10.035 20109743

[B59] Calderon MA Alves B Jacobson M Hurwitz B Sheikh A Durham S Allergen injection immunotherapy for seasonal allergic rhinitis Cochrane Database of Systematic Reviews 2007 1 CD001936 DOI: 10.1002/14651858.CD001936.pub2 10.1002/14651858.CD001936.pub2 PMC7017974 17253469

[B60] Radulovic S Calderon MA Wilson D Durham S Sublingual immunotherapy for allergic rhinitis Cochrane Database of Systematic Reviews 2010 12 CD002893 DOI: 10.1002/14651858.CD002893.pub2 10.1002/14651858.CD002893.pub2 PMC7001038 21154351

[B61] Abramson MJ Puy RM Weiner JM Injection allergen immunotherapy for asthma Cochrane Database of Systematic Reviews 2010 8 CD001186 DOI: 10.1002/14651858.CD001186.pub2 10.1002/14651858.CD001186.pub2 PMC13199908 20687065

[B62] Blumberga G Groes L Haugaard L Dahl R Steroid-sparing effect of subcutaneous SQ-standardised specific immunotherapy in moderate and severe house dust mite allergic asthmatics Allergy 2006 61 843 848 10.1111/j.1398-9995.2006.01088.x 16792582

[B63] Zielen S Kardos P Madonini E Steroid-sparing effects with allergen-specific immunotherapy in children with asthma: a randomized controlled trial J Allergy Clin Immunol 2010 126 942 949 10.1016/j.jaci.2010.06.002 20624650

[B64] Taegtmeyer AB Steurer-Stey C Spertini F Bircher A Helbling A Miedinger D Schafroth S Scherer K Leuppi JD Allergic rhinitis in patients with asthma: the Swiss LARA (Link Allergic Rhinitis in Asthma) survey Curr Med Res Opin 2009 25 1073 1080 10.1185/03007990902820733 19292600

[B65] Allen DB Effects of inhaled steroids on growth, bone metabolism and adrenal function Expert Rev Respir Med 2007 1 65 74 10.1586/17476348.1.1.65 20477267

[B66] Jacobsen L Primary and secondary endpoints in clinical trials Arb Paul Ehrlich Inst Bundesamt Sera Impfstoffe Frankf A M 2009 96 96 104 20799449

[B67] Pfaar O Kleine-Tebbe J Hormann K Klimek L Allergen-specific immunotherapy: which outcome measures are useful in monitoring clinical trials? Immunol Allergy Clin North Am 2011 31 289 309 x 10.1016/j.iac.2011.02.004 21530821

[B68] Hoiby AS Strand V Robinson DS Sager A Rak S Efficacy, safety, and immunological effects of a 2-year immunotherapy with Depigoid birch pollen extract: a randomized, double-blind, placebo-controlled study Clin Exp Allergy 2010 40 1062 1070 10.1111/j.1365-2222.2010.03521.x 20642579

[B69] Pfaar O Robinson DS Sager A Emuzyte R Immunotherapy with depigmented-polymerized mixed tree pollen extract: a clinical trial and responder analysis Allergy 2010 65 1614 1621 10.1111/j.1398-9995.2010.02413.x 20645937

[B70] Hafner D Reich K Matricardi PM Meyer H Kettner J Narkus A Prospective validation of 'Allergy-Control-SCORE(TM)': a novel symptom-medication score for clinical trials Allergy 2011 66 629 636 10.1111/j.1398-9995.2010.02531.x 21261656

[B71] Durham SR Birk AO Andersen JS Days with severe symptoms: an additional efficacy endpoint in immunotherapy trials Allergy 2011 66 120 123 10.1111/j.1398-9995.2010.02434.x 20608918

[B72] Varney VA Gaga M Frew AJ Aber VR Kay AB Durham SR Usefulness of immunotherapy in patients with severe summer hay fever uncontrolled by antiallergic drugs [see comments] BMJ 1991 302 265 269 10.1136/bmj.302.6771.265 1998791 PMC1668945

[B73] Varney VA Edwards J Tabbah K Brewster H Mavroleon G Frew AJ Clinical efficacy of specific immunotherapy to cat dander: a double-blind placebo-controlled trial Clin Exp Allergy 1997 27 860 867 10.1111/j.1365-2222.1997.tb01225.x 9291281

[B74] Dahl R Kapp A Colombo G de Monchy JG Rak S Emminger W Rivas MF Ribel M Durham SR Efficacy and safety of sublingual immunotherapy with grass allergen tablets for seasonal allergic rhinoconjunctivitis J Allergy Clin Immunol 2006 118 434 440 10.1016/j.jaci.2006.05.003 16890769

[B75] Didier A Malling HJ Worm M Horak F Jager S Montagut A de BO Andre C Melac M Optimal dose, efficacy, and safety of once-daily sublingual immunotherapy with a 5-grass pollen tablet for seasonal allergic rhinitis J Allergy Clin Immunol 2007 120 1338 1345 10.1016/j.jaci.2007.07.046 17935764

[B76] Drachenberg KJ Heinzkill M Urban E Woroniecki SR Efficacy and tolerability of short-term specific immunotherapy with pollen allergoids adjuvanted by monophosphoryl lipid A (MPL) for children and adolescents Allergol Immunopathol (Madr) 2003 31 270 277 10.1157/13052432 14572416

[B77] DuBuske LM Frew AJ Horak F Keith PK Corrigan CJ Aberer W Holdich T von Weikersthal-Drachenberg KJ Ultrashort-specific immunotherapy successfully treats seasonal allergic rhinoconjunctivitis to grass pollen Allergy Asthma Proc 2011 32 239 247 10.2500/aap.2011.32.3453 21535913

[B78] Balda BR Wolf H Baumgarten C Klimek L Rasp G Kunkel G Muller S Mann W Hauswald B Heppt W Tree-pollen allergy is efficiently treated by short-term immunotherapy (STI) with seven preseasonal injections of molecular standardized allergens Allergy 1998 53 740 748 9722222 10.1111/j.1398-9995.1998.tb03969.x

[B79] Klimek L Mewes T Wolf H Hansen I Schnitker J Mann WJ The effects of short-term immunotherapy using molecular standardized grass and rye allergens compared with symptomatic drug treatment on rhinoconjunctivitis symptoms, skin sensitivity, and specific nasal reactivity Otolaryngol Head Neck Surg 2005 133 538 543 10.1016/j.otohns.2005.07.020 16213926

[B80] Brehler R Kahlert H Thum Oltmer S Hypoallergenic preparations in SCIT. Immunological features and their impact on clinical efficacy and safety, exemplary for the allergoids Allergovit^®^, Acaroid^® ^and a folding variant of the recombinant birch pollen major allergen Bet v 1 Allergo Journal 2010 19 477 484

[B81] Jutel M Jaeger L Suck R Meyer H Fiebig H Cromwell O Allergen-specific immunotherapy with recombinant grass pollen allergens J Allergy Clin Immunol 2005 116 608 613 10.1016/j.jaci.2005.06.004 16159631

[B82] Hedlin G Graff Lonnevig V Heilborn H Lilja G Norrlind K Pegelow K Sundin B Lowenstein H Immunotherapy with cat- and dog-dander extracts. V. Effects of 3 years of treatment J Allergy Clin Immunol 1991 87 955 964 10.1016/0091-6749(91)90417-M 2026846

[B83] Haugaard L Dahl R Jacobsen L A controlled dose-response study of immunotherapy with standardized, partially purified extract of house dust mite: clinical efficacy and side effects J Allergy Clin Immunol 1993 91 709 722 10.1016/0091-6749(93)90190-Q 8454793

[B84] Pichler CE Marquardsen A Sparholt S Lowenstein H Bircher A Bischof M Pichler WJ Specific immunotherapy with Dermatophagoides pteronyssinus and D. farinae results in decreased bronchial hyperreactivity Allergy 1997 52 274 283 10.1111/j.1398-9995.1997.tb00991.x 9140517

[B85] O'Byrne PM Allergen-induced airway inflammation and its therapeutic intervention Allergy Asthma Immunol Res 2009 1 3 9 10.4168/aair.2009.1.1.3 20224664 PMC2831571

[B86] Rosewich M Schulze J Eickmeier O Telles T Rose MA Schubert R Zielen S Tolerance induction after specific immunotherapy with pollen allergoids adjuvanted by monophosphoryl lipid A in children Clin Exp Immunol 2010 160 403 410 10.1111/j.1365-2249.2010.04106.x 20345983 PMC2883111

[B87] Dahl R Kapp A Colombo G de Monchy JG Rak S Emminger W Riis B Gronager PM Durham SR Sublingual grass allergen tablet immunotherapy provides sustained clinical benefit with progressive immunologic changes over 2 years J Allergy Clin Immunol 2008 121 512 518 10.1016/j.jaci.2007.10.039 18155284

[B88] Dolz I Martinez-Cocera C Bartolome JM Cimarra M A double-blind, placebo-controlled study of immunotherapy with grass-pollen extract Alutard SQ during a 3-year period with initial rush immunotherapy Allergy 1996 51 489 500 8863926 10.1111/j.1398-9995.1996.tb04655.x

[B89] Niggemann B Jacobsen L Dreborg S Ferdousi HA Halken S Host A Koivikko A Koller DY Norberg LA Urbanek R Five-year follow up on the PAT study: Specific immunotherapy and long-term prevention of asthma in children Allergy 2006 61 855 859 10.1111/j.1398-9995.2006.01068.x 16792584

[B90] Jacobsen L Niggemann B Dreborg S Ferdousi HA Halken S Host A Koivikko A Norberg LA Valovirta E Wahn U Specific immunotherapy has long-term preventive effect of seasonal and perennial asthma: 10-year follow-up on the PAT study Allergy 2007 62 943 948 10.1111/j.1398-9995.2007.01451.x 17620073

[B91] Durham SR Emminger W Kapp A de Monchy JG Rak S Scadding GK Wurtzen PA Andersen JS Tholstrup B Riis B SQ-standardized sublingual grass immunotherapy: Confirmation of disease modification 2 years after 3 years of treatment in a randomized trial J Allergy Clin Immunol 2012 129 3 717 725 10.1016/j.jaci.2011.12.973 22285278

[B92] Mosbech H Osterballe O Does the effect of immunotherapy last after termination of treatment? Follow-up study in patients with grass pollen rhinitis Allergy 1988 43 523 529 10.1111/j.1398-9995.1988.tb01631.x 3148282

[B93] Jacobsen L Nuchel PB Wihl JA Lowenstein H Ipsen H Immunotherapy with partially purified and standardized tree pollen extracts. IV. Results from long-term (6-year) follow-up Allergy 1997 52 914 920 10.1111/j.1398-9995.1997.tb01251.x 9298176

[B94] Hedlin G Heilborn H Lilja G Norrlind K Pegelow KO Schou C Lowenstein H Long-term follow-up of patients treated with a three-year course of cat or dog immunotherapy J Allergy Clin Immunol 1995 96 879 885 10.1016/S0091-6749(95)70223-7 8543744

[B95] Des-Roches A Paradis L Knani J Hejjaoui A Dhivert H Chanez P Bousquet J Immunotherapy with a standardized Dermatophagoides pteronyssinus extract V Duration of the efficacy of immunotherapy after its cessation Allergy 1996 51 430 433 10.1111/j.1398-9995.1996.tb04643.x 8837669

[B96] Kettner J Mussler S Narkus A Häfner D Considerable 6 years post treatment long-term effect of pre-seasonal subcutaneous specific immunotherapy (SCIT) with a high-dose hypoallergenic grass pollen preparation. [abstract] Allergy 2011 66 296

[B97] Eng PA Reinhold M Gnehm HP Long-term efficacy of preseasonal grass pollen immunotherapy in children Allergy 2002 57 306 312 10.1034/j.1398-9995.2002.1o3264.x 11906360

[B98] Eng PA Borer-Reinhold M Heijnen IA Gnehm HP Twelve-year follow-up after discontinuation of preseasonal grass pollen immunotherapy in childhood Allergy 2006 61 198 201 10.1111/j.1398-9995.2006.01011.x 16409196

[B99] Novembre E Galli E Landi F Caffarelli C Pifferi M De ME Burastero SE Calori G Benetti L Bonazza P Coseasonal sublingual immunotherapy reduces the development of asthma in children with allergic rhinoconjunctivitis J Allergy Clin Immunol 2004 114 851 857 10.1016/j.jaci.2004.07.012 15480326

[B100] Johnstone DE Dutton A The value of hyposensitization therapy for bronchial asthma in children-a 14-year study Pediatrics 1968 42 793 802 5685362

[B101] Grembiale RD Camporota L Naty S Tranfa CM Djukanovic R Marsico SA Effects of specific immunotherapy in allergic rhinitic individuals with bronchial hyperresponsiveness [In Process Citation] Am J Respir Crit Care Med 2000 162 2048 2052 11112112 10.1164/ajrccm.162.6.9909087

[B102] Johnstone DE Crump L Value of hyposensitization therapy for perennial bronchial asthma in children Pediatrics 1961 61 44 13790388

[B103] Des-Roches A Paradis L Menardo JL Bouges S Daures JP Bousquet J Immunotherapy with a standardized Dermatophagoides pteronyssinus extract VI Specific immunotherapy prevents the onset of new sensitizations in children J Allergy Clin Immunol 1997 99 450 453 10.1016/S0091-6749(97)70069-1 9111487

[B104] Pajno GB Barberio G De Luca F Morabito L Parmiani S Prevention of new sensitizations in asthmatic children monosensitized to house dust mite by specific immunotherapy A six-year follow-up study Clin Exp Allergy 2001 31 1392 1397 10.1046/j.1365-2222.2001.01161.x 11591189

[B105] Purello-D'Ambrosio F Gangemi S Merendino RA Isola S Puccinelli P Parmiani S Ricciardi L Prevention of new sensitizations in monosensitized subjects submitted to specific immunotherapy or not A retrospective study Clin Exp Allergy 2001 31 1295 1302 10.1046/j.1365-2222.2001.01027.x 11529901

[B106] Marogna M Spadolini I Massolo A Canonica GW Passalacqua G Randomized controlled open study of sublingual immunotherapy for respiratory allergy in real-life: clinical efficacy and more Allergy 2004 59 1205 1210 10.1111/j.1398-9995.2004.00508.x 15461603

[B107] World Health Organization Prevention of Allergy and Allergic Asthma WHO report 2002

[B108] Allergic factors associated with the development of asthma and the influence of cetirizine in a double-blind, randomised, placebo-controlled trial: first results of ETAC. Early Treatment of the Atopic Child Pediatr Allergy Immunol 1998 9 116 124 10.1111/j.1399-3038.1998.tb00356.x 9814724

[B109] Bisgaard H Hermansen MN Loland L Halkjaer LB Buchvald F Intermittent inhaled corticosteroids in infants with episodic wheezing N Engl J Med 2006 354 1998 2005 10.1056/NEJMoa054692 16687712

[B110] Guilbert TW Morgan WJ Zeiger RS Mauger DT Boehmer SJ Szefler SJ Bacharier LB Lemanske RF Strunk RC Allen DB Long-term inhaled corticosteroids in preschool children at high risk for asthma N Engl J Med 2006 354 1985 1997 10.1056/NEJMoa051378 16687711

